# Endovascular Embolization of a Perforated Deep Femoral Artery in a 15-Year-Old Boy

**DOI:** 10.7759/cureus.33611

**Published:** 2023-01-10

**Authors:** Matthew H Bageris, Todd Chassee, Chris Benner

**Affiliations:** 1 Pediatric Emergency Medicine, Helen Devos Children's Hospital, Grand Rapids, USA; 2 Emergency Department, Michigan State University College of Human Medicine, Grand Rapids, USA

**Keywords:** trauma, pediatric, deep femoral, profunda femoris, penetrating stab wound, endovascular embolization

## Abstract

Trauma patients who are hemodynamically unstable or have certain signs of vascular injury should have immediate surgical exploration. For less severe signs of vascular injury, current literature states that endovascular intervention is optimal. This case presents the opportunity to review how signs of vascular injury were considered along with other diagnostic tools to inform decision-making after a penetrating stab wound injury to an extremity.

A 15-year-old male presented to the emergency department (ED) as a trauma activation after being stabbed in the left thigh. The patient had an approximately 5 cm long laceration over the lateral superior aspect of his left thigh with visible subcutaneous tissue and biceps femoris muscle upon probing. He had an initial blood pressure of 101/61 mm Hg. Shortly after the tourniquet was removed, the patient developed brisk bleeding from the wound and his blood pressure decreased to 88/55 mm Hg. He was taken expediently to computed tomography (CT) for an angiogram of the lower extremity which showed active bleeding from a posterior peripheral branch arising from the deep femoral artery in the posterolateral thigh. Interventional radiology performed intravascular embolization, and hemostasis was achieved. The patient was admitted for observation and then discharged 17 hours after admission without ambulatory difficulty.

We present a case of penetrating extremity trauma (PET) where the patient had a presentation with mixed hard signs and soft signs of vascular injury. The patient responded well to endovascular embolization. Early detection and intervention in PET are critical in minimizing blood loss and ischemia to distal structures. While following professional organization guidelines can help guide care, a collaborative approach by multiple specialty care teams is critical in providing optimal care in PET.

## Introduction

In civilian settings penetrating extremity traumas (PET) account for 5% to 15% of all trauma cases [1]. In these cases, vascular injuries account for a substantial portion of trauma-related deaths, with femoral and popliteal artery injuries accounting for approximately 50%-60% of deaths [2]. Of these injuries, 50% are caused by handguns and 30% are caused by stab wounds [3].

Identifying PET and associated vascular injury typically relies on recognizing “hard” and “soft” signs of vascular injury. The “hard signs” of vascular injury include the following criteria: pulsatile bleeding, absent distal pulses, cold/pale limbs, active hemorrhage and paresthesia. Patients with these “hard signs” warrant immediate vascular intervention. “Soft signs” of vascular injury include diminished pulses, close proximity to a major artery, and unexplained hypotension. In patients with these “soft signs,” recent literature has suggested doppler ultrasound as the initial diagnostic tool [3-5]. Further imaging or interventions can then be ordered based on these results.

In accordance with the guidelines placed by the Western Trauma Association and the Eastern Association for the Surgery of Trauma, endovascular embolization should be selectively applied to hemodynamically stable patients without hard signs of vascular injury and who have injuries to the lower extremity branch vessels [6]. Patients who are hemodynamically unstable or have hard signs of vascular injury should have immediate surgical exploration [7]. It is critical to decide which intervention is appropriate given the difference in morbidities and recovery time associated with open versus endovascular repair. This case presents the opportunity to review how signs of vascular injury should be used to inform decision-making and steps of intervention.

## Case presentation

**Figure 1:**
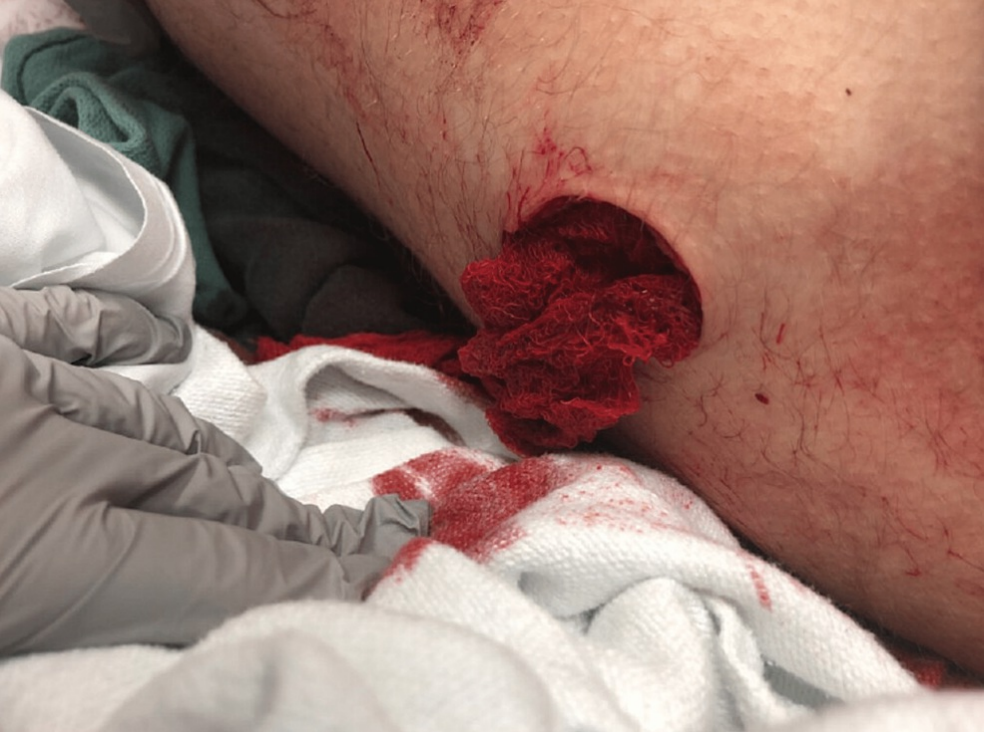
Five centimeters stab wound over the lateral superior aspect of the left thigh

A 15-year-old male presented to the emergency department (ED) as a trauma activation after being stabbed in the left thigh. He was involved in a fight where he was stabbed with a switchblade. The patient was brought by emergency medical services (EMS) 40 minutes after the event (Figure 1). Upon initial evaluation, his blood pressure was 101/61 mm Hg (left arm, supine, automated cuff) and heart rate was 107. The patient had an approximately 5 cm laceration over the lateral superior aspect of his left thigh with obvious, visible subcutaneous tissue and biceps femoris muscle upon probing. Other initial injuries included a 5 mm shallow laceration in the middle of his forehead and a 1 cm laceration over the distal aspect of the left fifth digit of the hand, with no exposed bone, muscle or tendon. A tourniquet was in place over the proximal left lower extremity and the left dorsalis pedis pulse was unobtainable by palpation but was biphasic on doppler. There was also a severely delayed capillary refill in the left foot. The patient reported severe pain throughout his left leg and he was unable to move his left foot. However, no paresthesia was noted upon physical examination.

**Figure 2:**
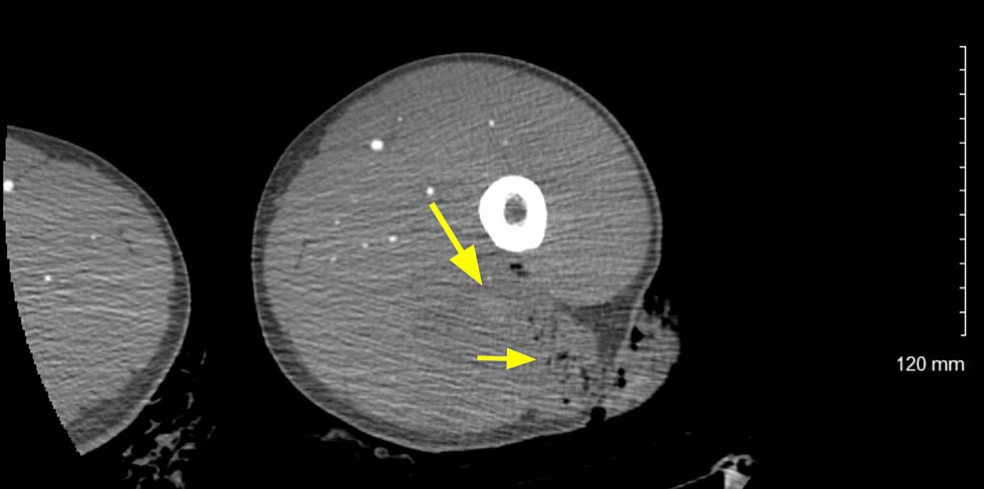
Axial CT angiogram The image shows air in the muscle (small arrow) and active extravasation of contrast (large arrow).

**Figure 3:**
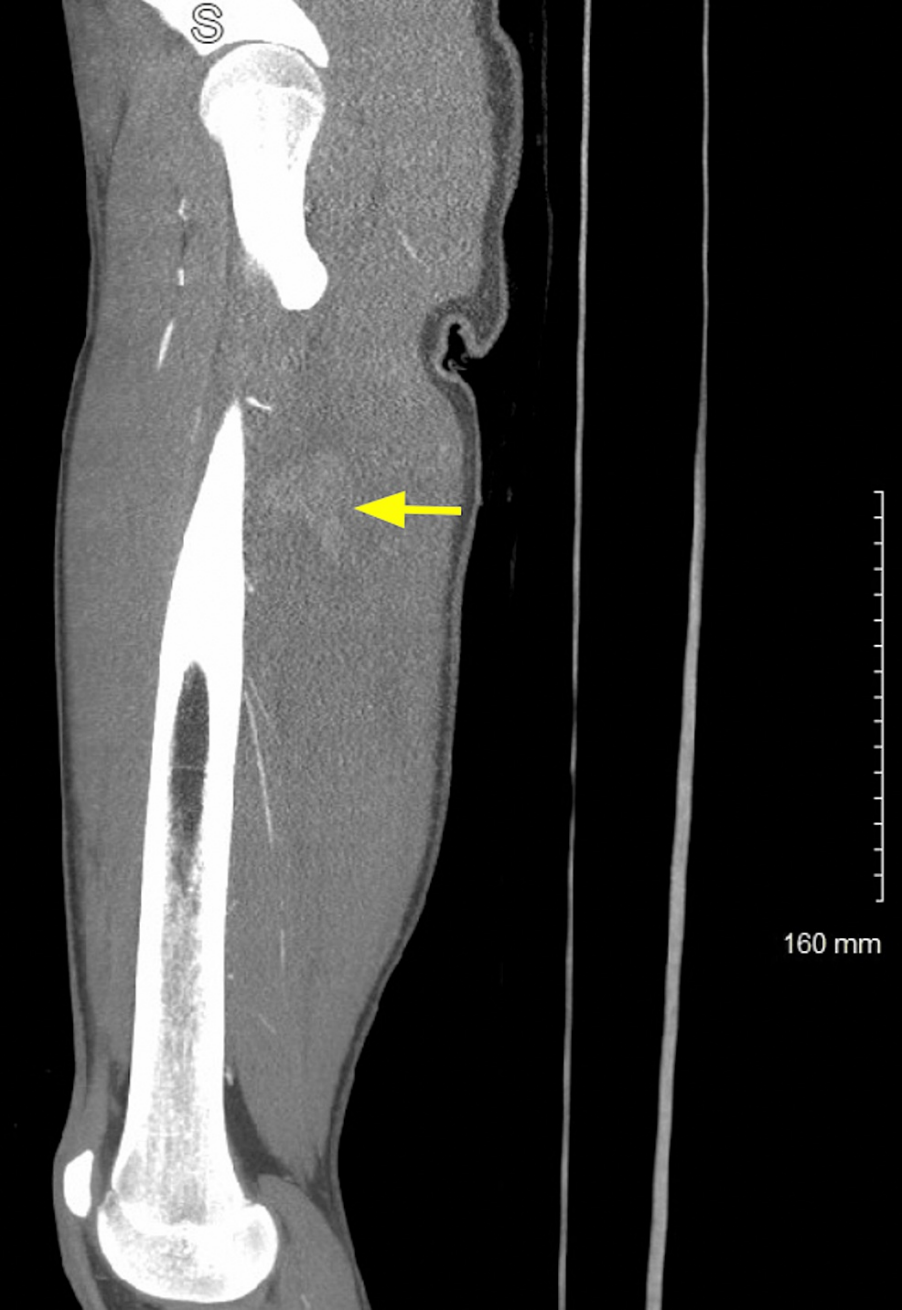
Sagittal CT angiogram The angiogram shows extravasation of contrast (arrow).

The patient was given 2 L of intravenous fluids and 50 μg of fentanyl for pain control. The tourniquet was removed and the patient had initial oozing of blood, which stopped spontaneously. The patient then had a return of a palpable dorsalis pedis pulse to palpation and he was able to actively range his foot with good strength. Shortly after, the patient developed brisk bleeding from the wound and his blood pressure decreased to 88/55 mm Hg. He was given two units of crossed platelet red blood cells (PRBC) and one unit of platelets. His blood pressure quickly stabilized. The wound was gently probed and was found to be deep. The trauma and vascular team could not obtain control of the hemorrhage at the bedside. He was taken expediently for computed tomography (CT) angiogram of the lower extremity which showed active bleeding from a posterior peripheral branch arising from the deep femoral artery in the posterolateral thigh (posterior branch of the profunda femoris) (Figures 2, 3).

The injury was deemed amenable to Interventional Radiology (IR) embolization after discussion with the attending trauma physician, vascular surgeon, and interventional radiologist. The patient was sedated and intubated, and a Foley was placed in the ED prior to transfer to the IR suite.

In the IR suite, the right common femoral artery was accessed with a 21-gauge micro-puncture needle under direct ultrasound guidance. Using the Seldinger technique, a five-French sheath was placed and attached to a heparinized, pressurized bag of normal saline.

**Figure 4:**
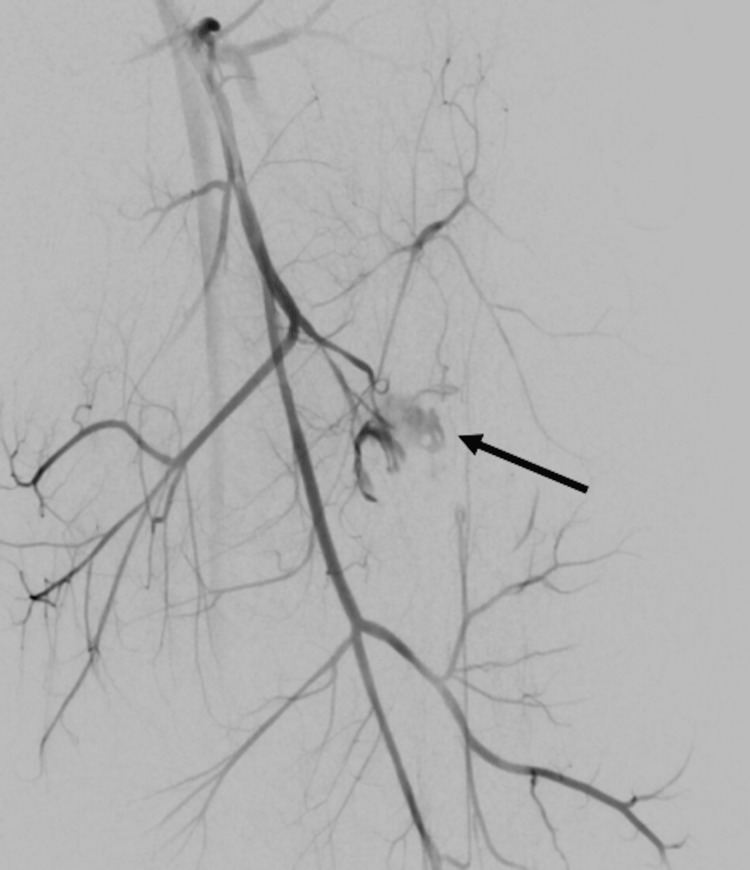
IR angiogram of the left profunda femoris The angiogram shows active extravasation (arrow). IR: Interventional Radiology

**Figure 5:**
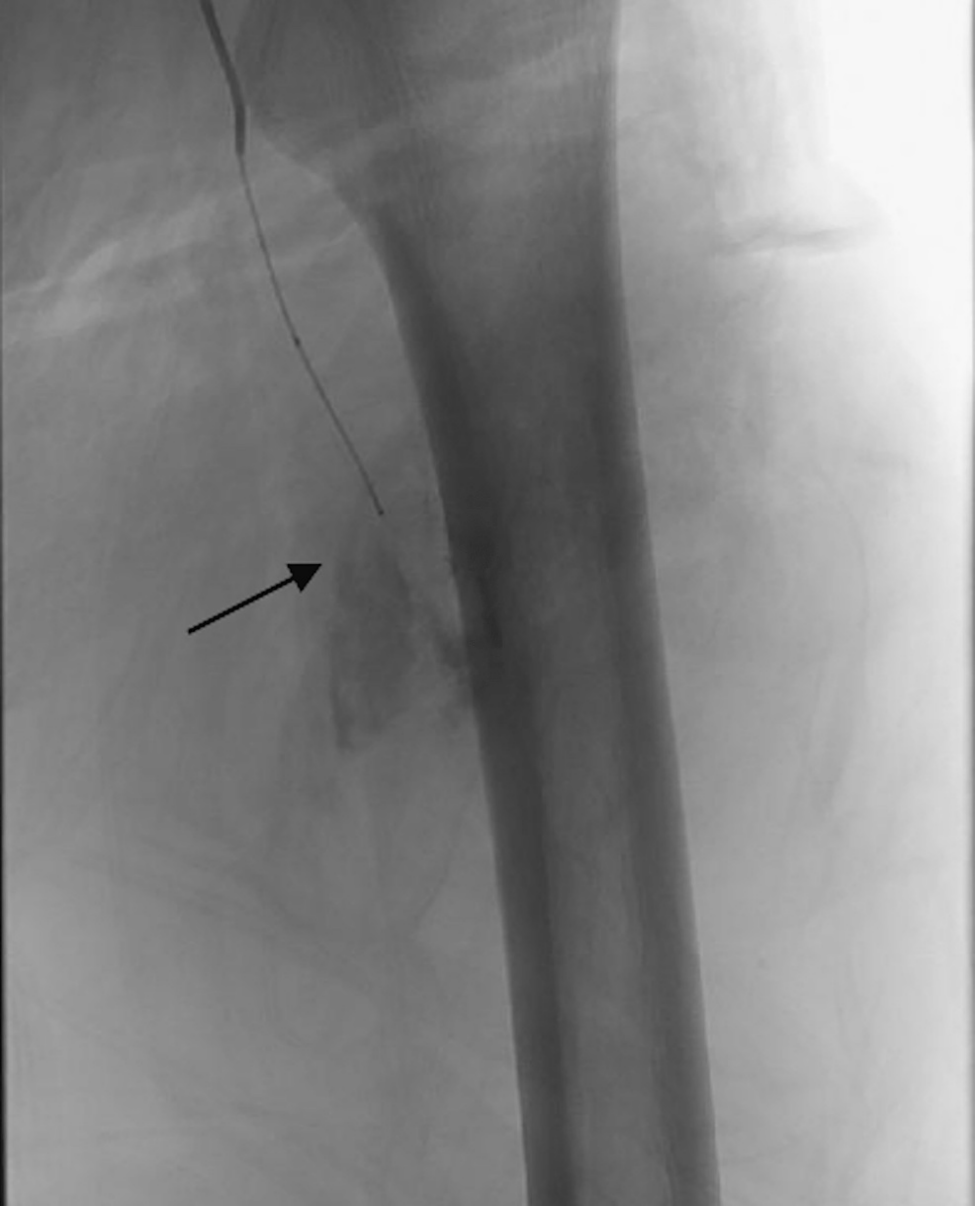
Initial IR injection into the profunda femoris The arrow shows active extravasation (arrow). IR: Interventional Radiology

**Figure 6:**
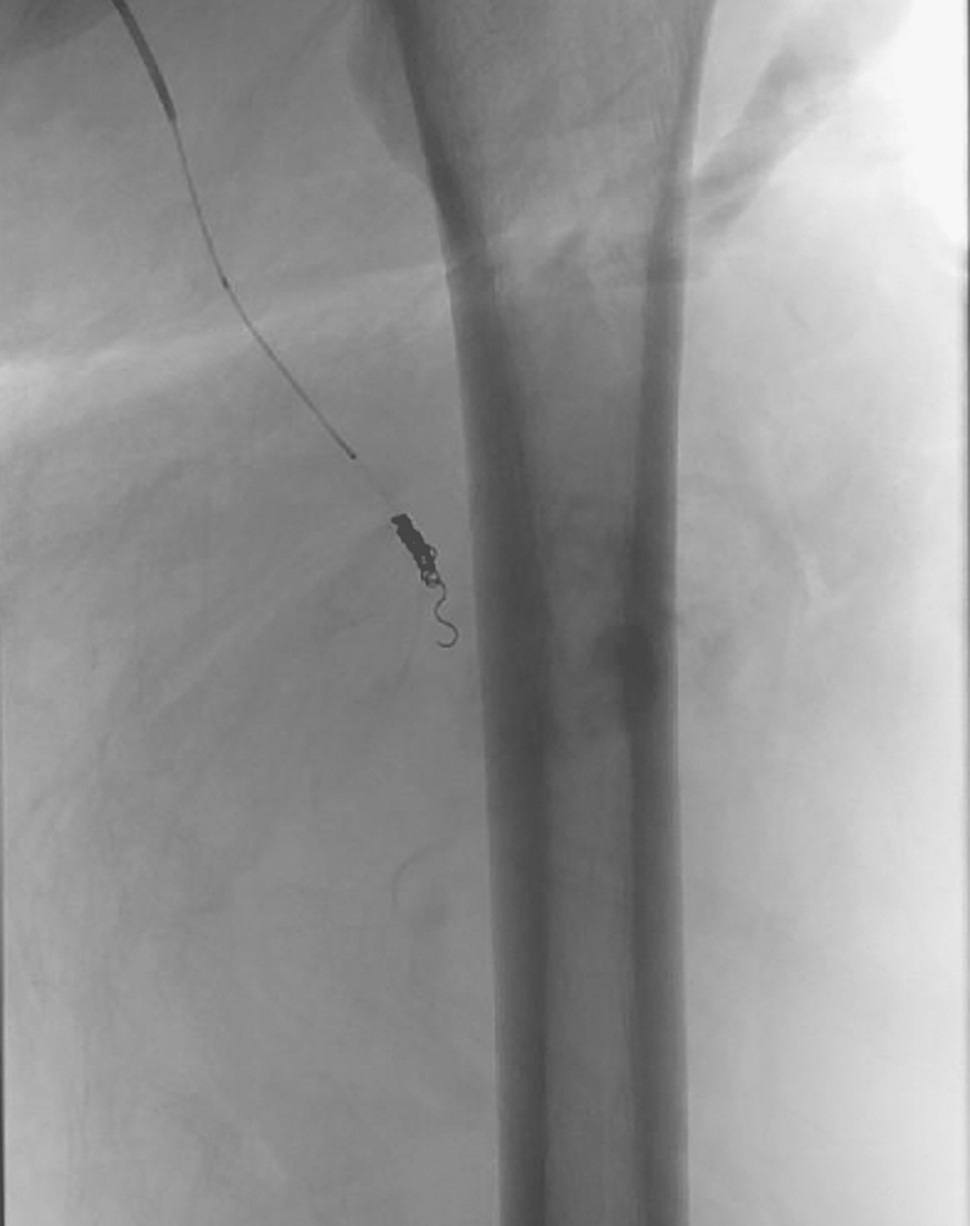
Initial coil placement

**Figure 7:**
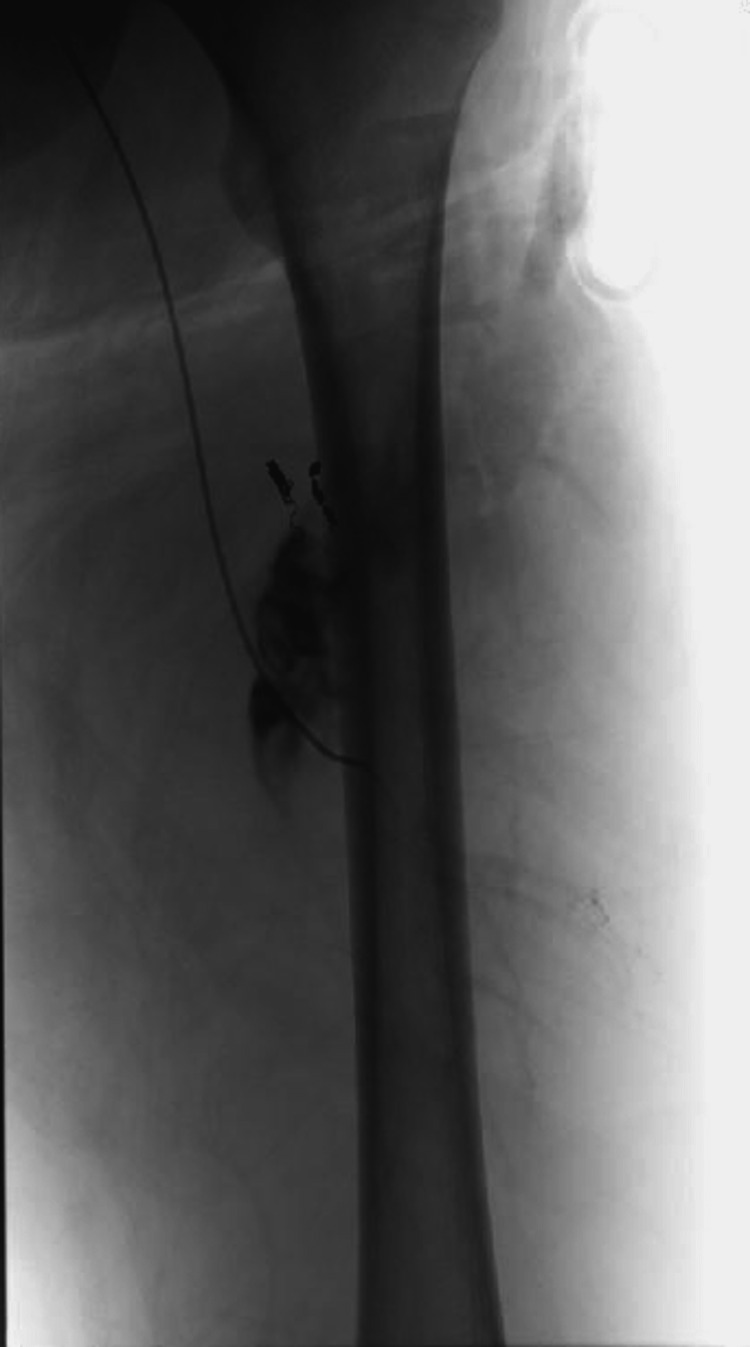
Injection prior to the second coil

**Figure 8:**
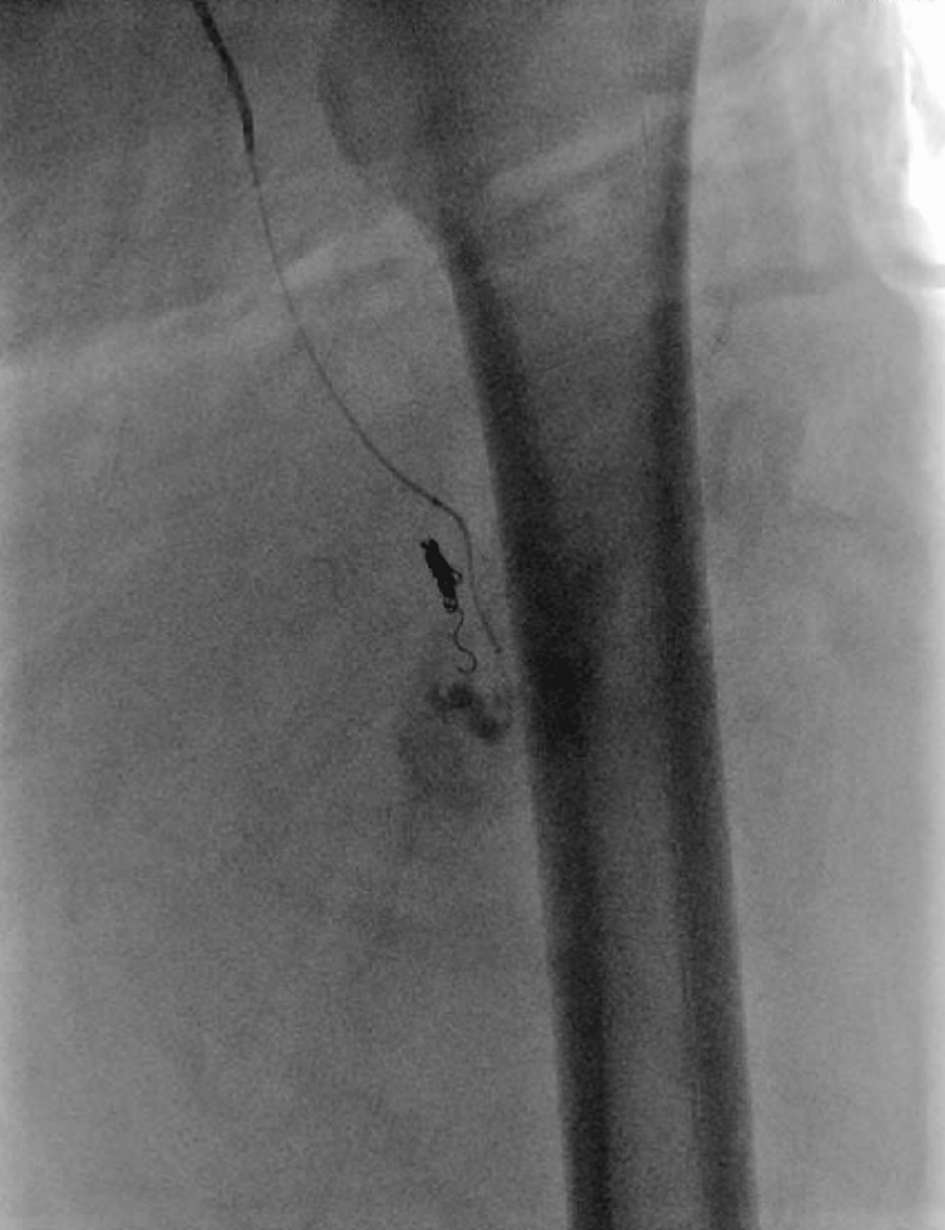
Injection after placement of the second coil

Next, a catheter and guidewire were introduced into the left common femoral artery (Figure 4) and an arteriogram was performed. The catheter and guidewire were placed into the profunda femoris and an arteriogram was performed. A microcatheter and microwire were advanced into the two separate branches of profunda femoris and corresponding arteriograms were performed (Figures 5-8). A selective arteriogram of the profunda femoral artery showed frank extravasation arising from the perforating branches in the mid-thigh corresponding to the site of the stab wound. A selective arteriogram of a perforating branch of the profunda femora showed that the vessel was truncated consistent with vascular injury.

Following embolization, there was no flow distal to the coil pack. An arteriogram of an adjacent perforating branch of the profunda femoris showed frank extravasation of contrast. Both branch vessels were embolized using micro-Nester coils. A follow-up arteriogram was performed. Catheters and wires were removed. Hemostasis was achieved with manual pressure after the sheath was removed and a sterile dressing was applied. The time to embolization from initial arrival at the ED was two hours and 50 minutes.

The wound site was then closed using seven 4-0 Ethilon simple interrupted sutures. The patient was then extubated and the Foley was removed. Lastly, the left fifth finger laceration was closed using three 5-0 Ethilon simple interrupted sutures and the forehead laceration was closed using liquid skin adhesive.

The patient was admitted for observation and he was hemodynamically stable with minimal oozing from the thigh wound on re-evaluation. Strong pedal pulses were noted with no numbness or paresthesia. He was discharged approximately 17 hours after admission and was ambulatory without difficulty at the time of discharge. After a two-week follow-up, the patient experienced no wound complications. The patient was asymptomatic aside from postural lightheadedness, which normalized after 30-60 seconds.

## Discussion

Initially, the patient had one worrisome, “hard sign” of vascular injury which was the absence of a dorsalis pedis pulse. Many of the more worrisome initial findings were resolved with the removal of the tourniquet. The dorsalis pedis pulse returned and his strength improved after the removal of the tourniquet. Although the patient experienced a brief period of bleeding once the tourniquet was removed, no excessive active hemorrhaging or pulsatile bleeding was present and his vital signs improved with fluid/PRBC resuscitation. The wound was initially thought not to involve large vascular structures due to a lack of active hemorrhage. The patient stated that he could not move his leg initially, but no paresthesia was mentioned.

This patient showed transient hard signs upon presentation. Ultimately, the care team decided embolization would be necessary as bedside exploration could not identify the source. He had an absent distal pulse, which resolved with the removal of the tourniquet. As mentioned above, endovascular embolization is often recommended for hemodynamically stable patients without hard signs. There were mixed hard and soft signs, most of which improved upon the removal of the tourniquet. He did develop an active hemorrhage with transient low blood pressure, which was controlled with direct pressure to the wound. Ultimately, this patient did well with endovascular embolization. It is important to note that with open repair, there is often a longer postoperative stay with a delay in ambulation compared to endovascular therapy [8]. In our case, he was discharged 17 hours after admission and was able to ambulate on his own.

In a similar case presentation by Blanco and Menéndez, a patient was stabbed, leaving a 3 cm laceration on the anterolateral aspect of the left thigh. Distal pulses were diminished, and leg numbness was noted by the patient. In this case, soft and hard signs were present. Point-of-care doppler ultrasound (DUS) was utilized, which showed a subfascial hematoma that was filled on color doppler. The patient was then immediately transferred to the operating room for surgical intervention [4].

Angiography is the gold standard for making the diagnosis of vascular injury. Angiography has a high sensitivity and specificity for arterial injury [9], with a low false positive rate [10]. Current practices and literature also view physical examination as a highly specific test for detecting arterial damage PETs [11].

This case demonstrates a scenario where arterial embolization was helpful despite initial intervention being delayed until an active hemorrhage developed. After control of the hemorrhage could not be obtained at bedside, a CT angiogram of the left lower extremity was then ordered and the extent of the arterial damage was realized. It is important that all facets of examination be taken into account to determine the necessary care plan for this patient. Collaborative healthcare team decision-making allowed for swift interventions after the patient became hypotensive and required resuscitation.

Various diagnostic modalities and clinical examination should be considered when patients present with PET. Quick intervention and collaborative decision-making at this case allowed for appropriate surgical intervention and a successful outcome despite an active hemorrhage with unstable vital signs.

## Conclusions

We presented a case of a PET where the patient had a presentation with mixed hard signs and soft signs of vascular injury. When bedside surgical repair was not possible, the patient responded well to endovascular embolization. Other aspects of clinical decision-making should be considered, such as physical examination or DUS. Early detection and intervention in PET are critical in minimizing hemorrhaging and further injury. While following professional guideline recommendations may be helpful, a collaborative approach by multiple specialty care teams, as displayed in this case, is critical in providing efficient and effective care for PET injuries. 
